# The Regenerative Capacity of Tissue-Engineered Amniotic Membranes

**DOI:** 10.1021/acsabm.3c00765

**Published:** 2024-02-23

**Authors:** Lennart Maljaars, Aksel Gudde, Anel Oosthuysen, Jan-Paul Roovers, Zeliha Guler

**Affiliations:** † Department of Obstetrics and Gynecology, Amsterdam UMC location University of Amsterdam, Meibergdreef 9, 1105 AZ Amsterdam, The Netherlands; ‡ Amsterdam Reproduction and Development research institute, Meibergdreef 9, 1105 AZ Amsterdam, The Netherlands; § Cardiovascular Research Unit, University of Cape Town, Anzio Road, Observatory, 7925 Cape Town, South Africa

**Keywords:** amniotic membrane, fibrin glue, poly-4-hydroxybutyrate (P4HB), scaffold, tissue engineering, vesicovaginal fistula

## Abstract

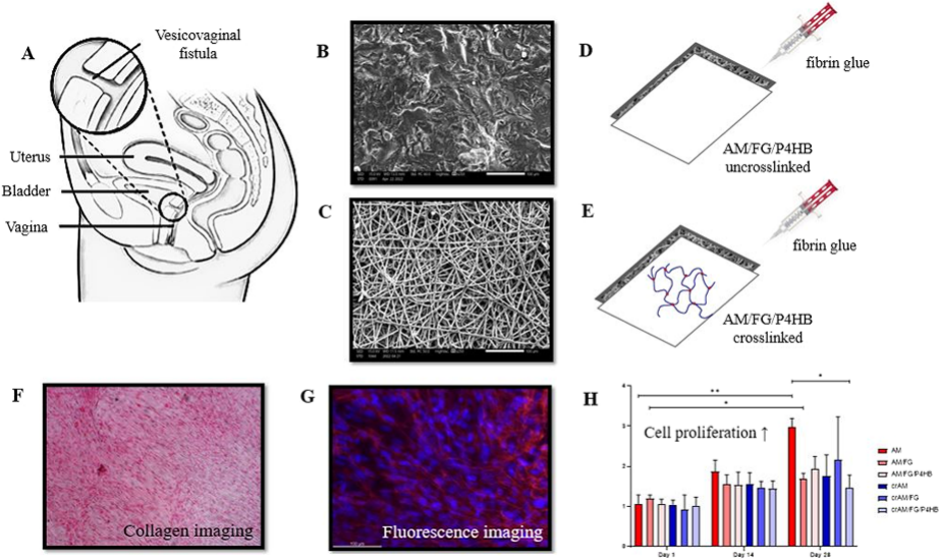

Scaffolds can be introduced as a source of tissue in reconstructive surgery and can help to improve wound healing. Amniotic membranes (AMs) as scaffolds for tissue engineering have emerged as promising biomaterials for surgical reconstruction due to their regenerative capacity, biocompatibility, gradual degradability, and availability. They also promote fetal-like scarless healing and provide a bioactive matrix that stimulates cell adhesion, migration, and proliferation. The aim of this study was to create a tissue-engineered AM-based implant for the repair of vesicovaginal fistula (VVF), a defect between the bladder and vagina caused by prolonged obstructed labor. Layers of AMs (with or without cross-linking) and electrospun poly-4-hydroxybutyrate (P4HB) (a synthetic, degradable polymer) scaffold were joined together by fibrin glue to produce a multilayer scaffold. Human vaginal fibroblasts were seeded on the different constructs and cultured for 28 days. Cell proliferation, cell morphology, collagen deposition, and metabolism measured by matrix metalloproteinase (MMP) activity were evaluated. Vaginal fibroblasts proliferated and were metabolically active on the different constructs, producing a distributed layer of collagen and proMMP-2. Cell proliferation and the amount of produced collagen were similar across different groups, indicating that the different AM-based constructs support vaginal fibroblast function. Cell morphology and collagen images showed slightly better alignment and organization on the un-cross-linked constructs compared to the cross-linked constructs. It was concluded that the regenerative capacity of AM does not seem to be affected by mechanical reinforcement with cross-linking or the addition of P4HB and fibrin glue. An AM-based implant for surgical repair of internal organs requiring load-bearing functionality can be directly translated to other types of surgical reconstruction of internal organs.

## Introduction

A vesicovaginal fistula (VVF) is an abnormal opening between the bladder and vagina that results in continuous involuntary leakage of urine, which severely affects the quality of life of patients.^1^ VVFs result from obstructed labor, pelvic surgery, or irradiation, where tissue ischemia and necrosis lead to fistula formation between hollow organs and affects around 2.0–3.5 million women worldwide.^1,2^ Surgical repair of VVF is challenging due to poor tissue quality, excessive scar tissue, and poor perfusion of the surrounding tissue, creating a suboptimal environment for wound healing. Closing the defect, and subsequent regaining of continence, is complicated by a lack of viable tissue to achieve tension-free closure, often compromising vaginal length, diameter, and bladder capacity. Reduction of vaginal length or diameter and bladder capacity may result in problems during intercourse, conception, and/or labor and micturition, respectively.^3−5^

Introduction of viable tissue, through surgical flaps or the use of biomaterials, can form a solution for surgical reconstruction in patients with extensive tissue loss. Tissue-engineered scaffolds can be introduced as a matrix for tissue ingrowth and can help to improve wound healing by providing a matrix on which cells can migrate, proliferate, and express physiological behavior. We identified amniotic membrane (AM) as a promising biomaterial for the reconstruction of VVF defects due to its good regenerative capacity, biocompatibility, gradual degradability, and availability.^6−8^ AM promotes fetal-like scarless healing, provides a supporting matrix—similar to the extracellular matrix (ECM)—and contains different bioactive molecules, such as growth factors, cytokines, and protease inhibitors.^8,9^

As unmodified AM is relatively weak, modifications of the biomaterial were previously studied (e.g., cross-linking, multilayers, combination with other biomaterials) in order to meet the load-bearing requirements of bladder and vaginal tissues in VVF repair.^10,11^ Combining AM with electrospun poly-4-hydroxybutyrate (P4HB) scaffold seemed the most promising option as P4HB meets mechanical demands for implantation and *in vivo* load-bearing, integrates well with vaginal tissue, and promotes proliferation and collagen deposition of vaginal fibroblasts. P4HB is a synthetic, degradable polymer that can be attached to AM with fibrin glue.^11−15^ In addition, electrospinning of P4HB allows for further modification of the mechanical characteristics to match the characteristics of the host tissue. The combined AM, P4HB, and fibrin form a bioactive, degradable, nontoxic matrix that facilitates cell adhesion, proliferation, and migration.^13,14,16,^

In this study, constructs consisting of an amniotic membrane (with or without cross-linking) and electrospun P4HB scaffold by using fibrin glue were fabricated to create a VVF implant that is highly regenerative, mechanically sufficient, and easy to handle. However, it is suggested that the regenerative capacity of the AM may be compromised by modifications such as cross-linking and addition of another material.^18−20^ Therefore, the regenerative capacity of vaginal fibroblasts seeded on VVF implants was evaluated in this *in vitro* study, as vaginal fibroblasts are the main cell type of pelvic tissue and responsible for the ECM metabolism in wound healing. We used vaginal fibroblasts derived from patients with pelvic organ prolapse (POP). POP tissue may be considered as diseased tissue and therefore may better represent our disease model—obstetric fistula—which results from repetitive ischemic/crush injury.^21^ The *in vitro* outcome measurements relevant for wound healing were studied: cell proliferation, cell morphology, collagen deposition, and metabolism measured by matrix metalloproteinase (MMP) activity to assess the suitability of the AM-based implant for surgical repair of VVF.

## Materials and Methods

### Materials

Human amniotic membranes (CellReGen, Salt Lake City, UT) were used in all experiments. All donors were screened for infectious diseases prior to the harvest of the membranes. All samples were in sterile and dry condition. Poly-4-hydroxybutyrate (Tepha Inc., Cambridge, MA), a fully biodegradable polymeric biomaterial, was used to produce electrospun scaffolds, as described before.^11^ In short, P4HB was dissolved in chloroform and dimethylformamide (9:1 vol/vol) to form a 10% (w/w) solution. Electrospinning was performed with a custom-built rig. The polymer solution was supplied from a syringe pump via a positively charged spinneret to a negatively charged, adjustable speed rotating collector. AM was mounted on the P4HB scaffold with fibrin glue (Tisseel, Baxter, Utrecht, The Netherlands) using a spraying device. The fibrin glue was prepared in accordance with the instructions for use.

### Experimental Groups and Sample Preparation

The AM-based VVF implant was constructed as described previously.^11^ Both un-cross-linked AM (AM) and cross-linked AM (crAM) were used for the preparation of the constructs. Unmodified, un-cross-linked AM (AM) was used as the control group. The AM/FG group consisted of AM modified with a thin layer of fibrin glue. AM/FG/P4HB was created by combining AM and electrospun P4HB scaffolds with a layer of fibrin glue. The same construct constitutions were obtained by using AM cross-linked with 1% glutaraldehyde (GA) and γ-irradiated in the production process (crAM, crAM/FG, and crAM/FG/P4HB). The construction of the AM-based VVF implants and the mechanical properties (see Supporting Table 1), as well as the properties of the P4HB scaffold (pore size, fiber size, and scanning electron microscopy (SEM) images), have been described before.^11^

## Experimental Section

### Cell Culture

Primary human vaginal fibroblasts were isolated from a full-thickness vaginal biopsy, which was harvested during vaginal surgery for pelvic organ prolapse in a consenting patient.^13^ Vaginal fibroblasts were expanded until passage 3 and then seeded on the constructs.

Two different construct sizes were used: 0.8 × 0.8 cm^2^ samples were placed in 48 well plates (for cell proliferation, collagen deposition, colorimetry, and MMP activity) and 1.2 × 1.2 cm^2^ samples were placed in 24 well plates (for collagen imaging and fluorescence imaging). Scaffolds were placed with the AM-side facing upward, and custom-made glass rings (0.8 cm Ø) were used to prevent them from floating. All constructs were sterilized in 70% ethanol for 30 min and washed three times with phosphate-buffered saline (PBS) before cell seeding. Vaginal fibroblasts were seeded onto the different constructs with a density of 1.0 × 10^4^ cells/cm^2^ and cultured in Dulbecco’s modified Eagle’s medium (DMEM) (Gibco-Life Technologies, Paisley, U.K.) supplemented with 10% v/v fetal bovine serum (FBS) (HyClone, South Logan, UT), 100 mg/mL streptomycin, 100 U/ml penicillin (pen/strep), and 250 mg/mL amphotericin-B (Sigma, St. Louis, MO) at 37 °C and 5% CO_2_ in a humidified environment for 28 days.^22^ The medium was refreshed every 2–3 days. All experiments were performed on samples in triplicate and repeated three times.

### Vaginal Fibroblast Proliferation

The proliferation of vaginal fibroblasts was assessed with Alamar blue (Thermo Fisher) colorimetric viability assay. The constructs were washed with PBS at culture days 1, 14, and 28. Fibroblasts in each well were incubated with 250 μL 10% Alamar blue working solution (10% v/v Alamar blue in DMEM) for 3 h in the dark at 37 °C and 5% CO_2_. Metabolically active cells reduce resazurin (7-hydroxy-3H-phenoxazin-3-one 10-oxide) to resorufin. The Synergy HT multimode microplate reader (Biotek Instruments Inc., VT) was used to measure the absorbance of resazurin excreted by inactive cells (600 nm) and resorufin (570 nm) excreted by active cells.^23^ Proliferation was determined by calculating the difference in absorbance at 570 and 600 nm in Alamar blue supplemented conditioned medium.

### Cell Morphology

Cell morphology was assessed with fluorescence staining after 14 and 28 days of culture. Cells were fixated with 4% paraformaldehyde (PFA) for 15 min at room temperature and permeabilized in 5% Triton X-100 in PBS. The fibroblast actin filaments were stained with Acti-Stain 555 Phalloidin (Cytoskeleton, Inc.) for 30 min in the dark at room temperature. Nuclear counterstaining was performed with DAPI (4′,6-diamidino-2-phenylindole) (Life Technologies). A fluorescence microscope (Nikon Eclipse Ti–S with epifluorescence attachment) was used to obtain images after washing with PBS.^24^

### Collagen Deposition

Collagen deposition was assessed by Picro-Sirius Red staining, which has selective binding to collagen and semiquantitatively measured by colorimetry.^25^ Fixed cells at culture day 14 and 28 were rinsed with PBS twice and stained with 100 μL of 0.1% Sirius Red solution (Chondrex, Inc., WA) in picric acid. The dye was removed after 30 min, and samples were rinsed with PBS twice. For quantitative measurement, dye extraction buffer (Chondrex, Inc., WA; 1 mL) was added and mixed to elute the color from the cells. A multimode microplate reader was used to measure the absorbance of released Sirius Red (605 nm). The amount of collagen was calculated according to the manufacturer’s instructions.^14^ Samples for imaging of the deposited collagen were prepared in the same way on separate microscopy slides, with an additional 100% ethanol wash. The cells were observed with light microscopy at ×10–40 magnification.

### MMP Activity

Matrix metalloproteinases are a group of proteolytic enzymes that degrade ECM components and play an important role in ECM remodeling.^21^ Matrix metalloproteinase 2 (proMMP-2) activity was determined with gelatin zymography on day 14 and 28. Samples were refreshed with DMEM supplemented with pen/strep (no FBS) 1 day before the respective time points and stored at −80 °C for further processing. The conditioned medium was diluted with distilled water in a 1:3 ratio and pipetted into the wells of casted gelatin gels.^26^ Two wells were pipetted with a protein standard (Precision Plus Protein Dual Color Standard, Bio-Rad) and 1 ng collagenase for the identification of the MMP-2 subtype and for normalization, respectively. Gel electrophoresis was performed to separate MMPs based on molecular size. The gels were renatured and developed overnight.^27^ The gels were stained with SimplyBlue to uncover the digested gelatin bands (Thermo Scientific). proMMP-2 band intensity was quantified using ImageJ 1.53c and normalized to 1 ng collagenase.^28^

### Statistical Analysis

All data were tested for normality. Normally distributed data is reported as mean and standard deviation (SD), and non-normally distributed data is reported as mean and 25th and 75th percentile. Analyses were performed using Prism Graphpad version 9.0 (GraphPad Software, San Diego, CA). Groups of interest were compared with ANOVA, followed by Tukey–Kramer’s post hoc *t*-test (two-tailed) or Kruskall–Wallis test, followed by Dunn’s test. Repeated measurement ANOVA or Friedman’s test was used for repeated measurements, also followed by Tukey–Kramer’s post hoc test. A *p*-value of less than 0.05 was considered statistically significant.

## Results

### Vaginal Fibroblast Proliferation

**Figure 1:**
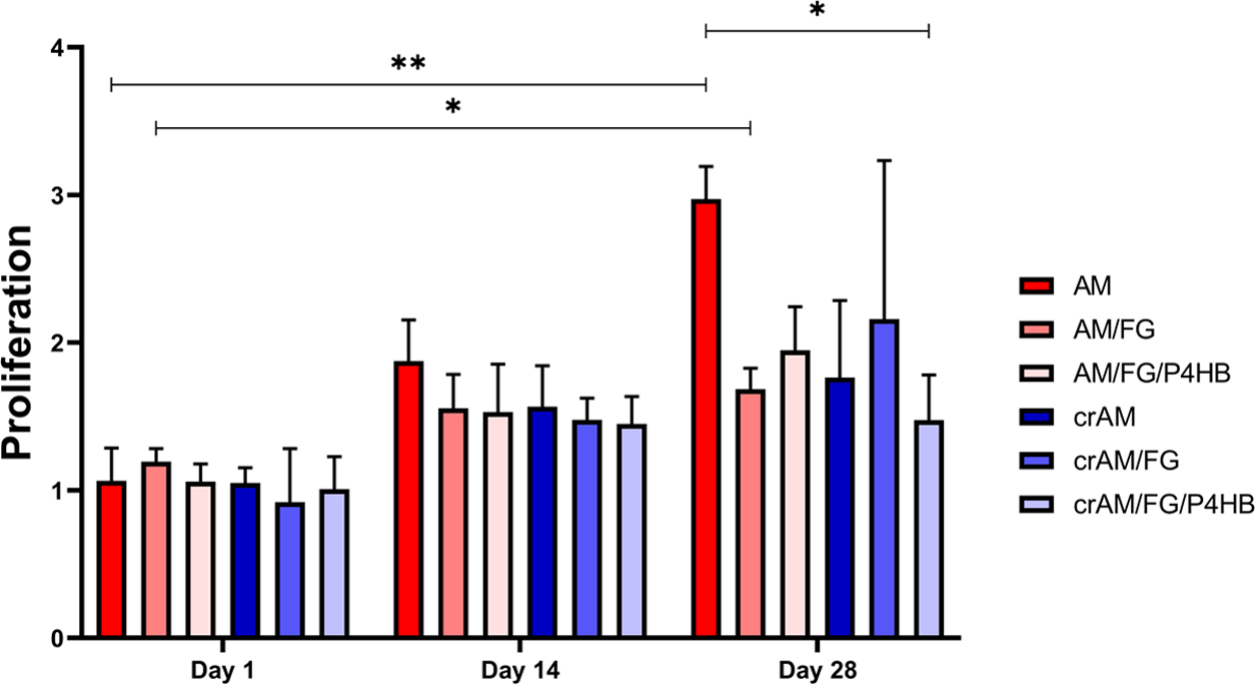
Proliferation vaginal fibroblasts. Proliferation of vaginal fibroblast measured by Alamar blue assay at culture day 1, 14, and 28. AM: un-cross-linked amniotic membranes; AM/FG: un-cross-linked amniotic membranes with fibrin glue; AM/FG/P4HB: un-cross-linked amniotic membranes with fibrin glue and electrospun poly-4-hydroxybutyrate (P4HB); crAM: cross-linked amniotic membranes; crAM/FG: cross-linked amniotic membranes with fibrin glue; crAM/FG/P4HB: cross-linked amniotic membranes with fibrin glue and electrospun poly-4-hydroxybutyrate (P4HB). *p < 0.05; **p < 0.01.

Un-cross-linked AM showed significantly better proliferation, measured by the absorbance of Alamar blue, compared to the cross-linked AM/FG/P4HB (cr/AM/FG/P4HB) at time point day 28 (Figure 1). Proliferation did not differ between the other experimental groups at each time point. There was an increase in cell proliferation from day 1 to day 28 in the AM and AM/FG group. In all other groups, there was no difference in proliferation over time. The crAM/FG performed better in one of the experimental rounds, which resulted in a larger range indicated by the error bar. This effect was not seen in the other rounds of the experiment.

### Cell Morphology

**Figure 2:**
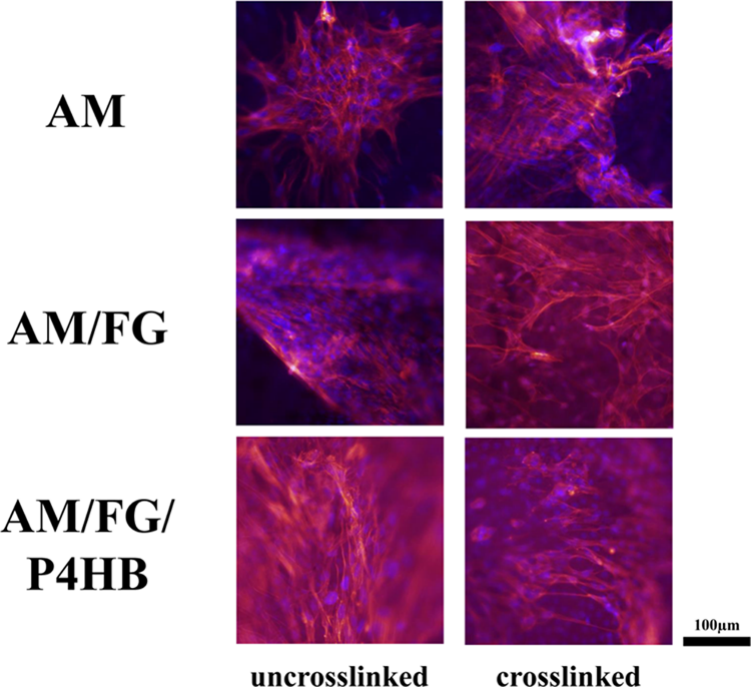
Fluorescent imaging immunofluorescence of vaginal fibroblast at culture day 14.

Fluorescent images of vaginal fibroblast morphology on day 14 revealed that vaginal fibroblasts attached and proliferated on the constructs (Figure 2). Proliferating fibroblasts were organized in clusters after 14 days with few interconnections between clusters of cells. The cytoskeletons of the clustered cells spread out with a random orientation on day 14. At day 28, the fibroblasts covered the complete surface of the constructs, which was especially visible on the constructs with un-cross-linked AM (Figure S1). The morphology of the cells at day 28 well matched with the ones at day 14, and alignment of the fibroblasts increased between day 14 and 28. The constructs with cross-linked amnion showed less coverage of the surface at day 28.

### Collagen Deposition

**Figure 3:**
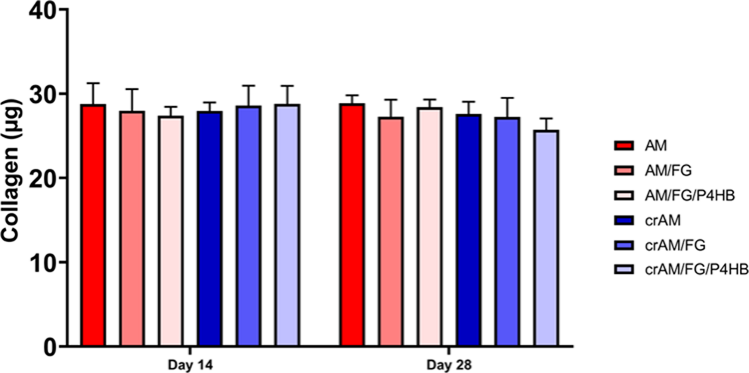
Collagen assay. Collagen amount in microgram (μg) on culture day 14 and 28. AM: un-cross-linked amniotic membranes; AM/FG: un-cross-linked amniotic membranes with fibrin glue; AM/FG/P4HB: un-cross-linked amniotic membranes with fibrin glue and electrospun poly-4-hydroxybutyrate (P4HB); crAM: cross-linked amniotic membranes; crAM/FG: cross-linked amniotic membranes with fibrin glue; crAM/FG/P4HB: cross-linked amniotic membranes with fibrin glue and electrospun poly-4-hydroxybutyrate (P4HB).

**Figure 4:**
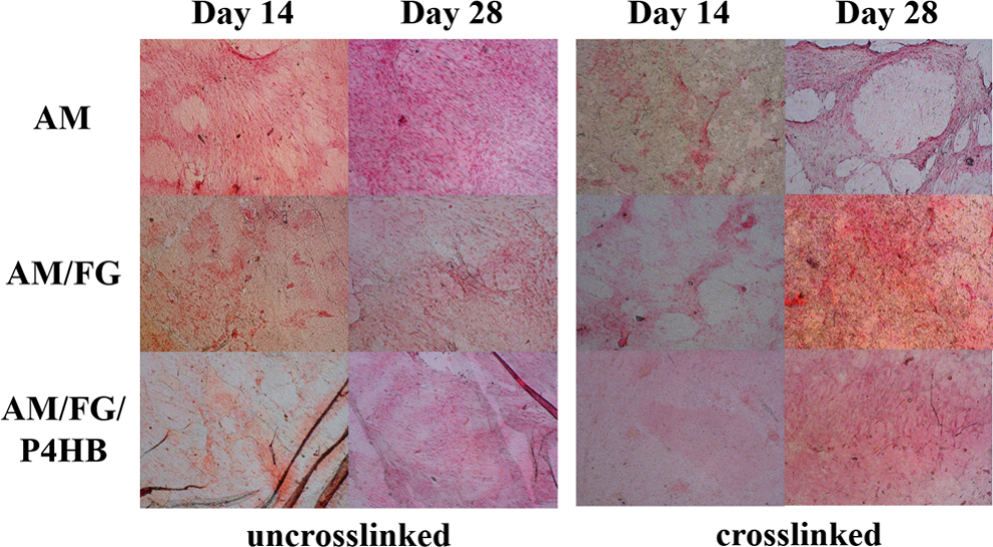
Collagen imaging. Collagen imaging at culture day 14 and 28 at ×20 magnification.

Assessment of collagen deposition on day 14 and 28 showed that the total amount of collagen (∼30 μg) was comparable across the different groups and time points (Figure 3). Addition of fibrin glue or P4HB did not change the amount of collagen produced by the vaginal fibroblasts. Bright-field images of deposited collagen stained with Picro-Sirius Red indicated increased collagen densities adjacent to vaginal fibroblast clusters after 14 days (Figure 4). Collagen aligned with vaginal fibroblasts near elongated cells that interconnected the clusters. This pattern was especially visible in the AM group. Deposited collagen spread over the construct surface and concentrated adjacent to the fibroblasts, which covered the construct’s surface with a confluent monolayer visible after 28 days. The constructs with un-cross-linked AM showed almost a complete coverage of the surface. The cross-linked samples showed a more irregular coverage with collagen connections between the different fibroblast clusters, as was seen on day 14 in the un-cross-linked groups.

### MMP Activity

**Figure 5:**
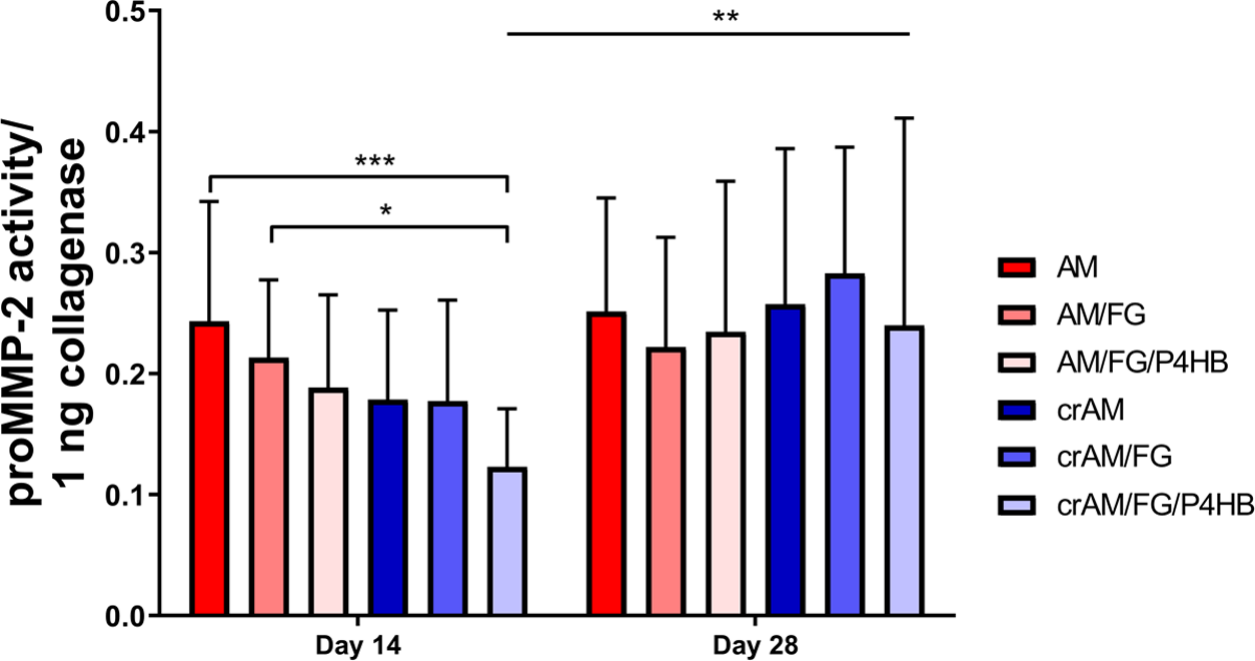
MMP activity. Matrix metalloproteinase (proMMP-2) activity on culture day 14 and 28. AM: un-cross-linked amniotic membranes; AM/FG: un-cross-linked amniotic membranes with fibrin glue; AM/FG/P4HB: un-cross-linked amniotic membranes with fibrin glue and electrospun poly-4-hydroxybutyrate (P4HB); crAM: cross-linked amniotic membranes; crAM/FG: cross-linked amniotic membranes with fibrin glue; crAM/FG/P4HB: cross-linked amniotic membranes with fibrin glue and electrospun poly-4-hydroxybutyrate (P4HB). *p < 0.05; **p < 0.01; and ***p < 0.001.

The proMMP-2 activity on day 14 decreased significantly for crAM/FG/P4HB as compared to the AM and AM/FG constructs (Figure 5). At day 28, the proMMP-2 activity was similar between all groups. Conditioned medium on the crAM/FG/P4HB construct exhibited a higher proMMP-2 activity at day 28 compared to day 14. An example of the zymography gel electrophoresis of proMMP is shown in Figure S2.

## Discussion

### Summary of Findings

In this study, we assessed cell response of vaginal fibroblasts on different AM-based constructs. Vaginal fibroblasts proliferated and were metabolically active on all constructs, producing a distributed layer of collagen and proMMP-2, which are relevant factors in functional ECM remodeling. Cell proliferation and the amount of produced collagen were similar across different groups, indicating that the different AM-based VVF implants support vaginal fibroblast function. Cell morphology and collagen images showed slightly better alignment and organization on the un-cross-linked constructs compared to the cross-linked constructs. In summary, the regenerative capacity of AM does not seem to be affected by mechanical reinforcement of AM with cross-linking or by the addition of fibrin glue and P4HB scaffold.

### Rationale for This Work and Previous Research

This study is part of a bigger effort to create a tissue-engineered AM-based implant for the surgical treatment of vesicovaginal fistula (VVF) repair. In our approach, we aimed to modify amniotic membranes to gain sufficient mechanical properties to withstand pressures and forces in hollow organs.^10,29^ We previously showed that chemical cross-linking alone does not yield sufficient mechanical strength for surgical implantation of AM.^11^ Amniotic xenograft transplantation has been demonstrated in different urological small animal models, with negligible inflammation and rejection response and complete epithelialization of the membranes.^30−34^ Only two case reports have reported on the clinical application of AM for VVF repair, with only one study applying it as a scaffold instead of an interposition patch.^35,36^ The study where multilayered AM was used reported a recurrence after 8 months, highlighting the need for mechanical reinforcement of amniotic membranes when applied for a bigger defect. In our previous study, we fabricated a construct with AM and P4HB combined with fibrin glue that showed sufficient mechanical characteristics for surgical implantation for vesicovaginal fistula repair.^11^

### Interpretation of Findings

The major concern is that modification of AMs might negatively affect the regenerative capacity since alterations could interfere with cell attachment and proliferation, and added chemical components may be cytotoxic. When combined with fibrin glue, or fibrin glue and P4HB, the proliferation and attachment of the fibroblasts on the AM was comparable with unmodified AM and between groups. Although vaginal fibroblast proliferation increased on AM as compared to the other constructs on day 28, the increase of proliferation over time was not affected by the modifications. This is in line with previous studies on the (electrospun) P4HB that show good cell attachment and proliferation of vaginal fibroblasts on P4HB and no signs of cytotoxicity.^14,15,13^ In addition, our results confirm that fibrin glue is also noncytotoxic.^16^ The fluorescence imaging in this study showed good cell adherence to the construct and spreading on the construct over time. This is in accordance with studies that demonstrate that all three materials used in the construct (AM, fibrin glue, P4HB) can form a bioactive matrix that stimulates cell adhesion, proliferation, and differentiation.^14−15,16,17^ In this study, we compared constructs made from un-cross-linked AMs and AMs that were previously cross-linked in 1% GA. Cross-linking can be applied to mechanically reinforce AMs and to increase resistance to proteolytic degradation of the tissue.^10^ However, glutaraldehyde can be cytotoxic depending on the concentration and duration of cross-linking.^18^ Our results show no significant effect of cross-linking on the metabolic activity of vaginal fibroblast represented by Alamar blue assay on the AM construct. The fluorescence and collagen imaging seemed to show better alignment and orientation of the vaginal fibroblasts and collagen fibrils after 28 days. The differences in alignment may result from a small cytotoxic effect of glutaraldehyde cross-linking that causes alteration in cell morphology and a decrease in cell viability. In addition, cross-linking can result in alteration of pore size, porosity, and fiber diameter, which can lead to a decrease in cell adhesion migration and proliferation. However, it did not lead to significant differences in cell proliferation, collagen amount, and MMP activity. The collagen amount was similar across groups and time points. In addition, potential ECM remodeling by MMP-2activity did not indicate an effect between groups that solely differed in terms of the presence of a cross-linker. This indifference seems to support the idea that cross-linking with 1% glutaraldehyde does not significantly affect the remodeling response of AMs.

Literature shows that the cytotoxicity of cross-linking with glutaraldehyde is dose- and time-dependent. Longer duration of cross-linking and higher concentration of cross-linking agents results in better resistance to degradation but is less well-tolerated by cell culture.^18−20^

The vaginal fibroblasts produced collagen on the AM-based VVF implants. Images showed a confluent monolayer of vaginal fibroblast and collagen fibrils after 28 days. Fibroblasts play a critical role in wound healing and tissue regeneration by production of collagen and remodeling of the ECM. Collagen I and III form the main components of the ECM of the pelvic floor and determine tissue strength.^21^ Collagen deposition strengthens the tissue and creates an ECM for other cells to grow and proliferate. This is balanced with collagen degradation as part of remodeling that is controlled by matrix metalloproteinases such as MMP-2.^21^ In this study, collagen deposition and proMMP-2 activity were similar among groups, which shows that modification of AM does not result in altered ECM regulation and remodeling. The detected increase in proMMP-2 on AM as compared to AM/FG/P4HB on day 14 was in line with an increasing trend in proliferation between the respective constructs. The alignment suggests a contribution of increased cell number to MMP-2 activity rather than increased remodeling activity by fibroblasts individually. A meta-analysis on the application of AM in reconstructive surgery showed no or a minimal foreign body response, low inflammation scores, and good epithelialization when the AM was applied in different human and animal models.^6^ This is supported by preclinical research that shows that AM exhibits anti-inflammatory,^37−39^ immunosuppressive,^40−42^ antifibrotic,^43,44^ antimicrobial,^45^ and immunomodulatory properties.^8,40,46−48^ Implantation of P4HB in a subcutaneous rat model showed a host response to the implant compared to sham surgery.^49^ In a sheep model, the host response was moderate, with good tissue integration and dense connective tissue, following vaginal implantation of P4HB.^14^ Although these results are promising, further evaluation in a more complex *in vivo* environment of an animal model should determine if the ECM remodeling on the VVF implant is well-balanced and results in functional healing.

In this study, P4HB was used for the mechanical reinforcement of the AM constructs as P4HB has been shown to exhibit sufficient mechanical characteristics and biocompatibility as a substrate *in vitro* and as an implant in different large animal models.^11,14,50^ P4HB has been shown to be able to gradually transfer load with balanced construct degradation and tissue regeneration.^18,51^ Other degradable biomaterials applied for pelvic floor repair such as polycaprolactone (PCL), polylactic-*co*-glycolic acid (PLGA), poly(lactic acid) (PLA), silk fibroin, and electrochemically aligned collagen have also shown promising results when studying the effect on vaginal fibroblast proliferation and ECM formation and may further expand the tissue engineering options to optimize the VVF implant for application.^21,52^

### Limitations

In this study, the growth factors, cytokines, and protease inhibitors that are hypothesized to be responsible for the regenerative capacity of AM were not analyzed. Some studies suggest that these bioactive components of AM can be lost or retained, depending on the handling procedures of the AM.^53^ Therefore, it remains to be determined to what extent bioactive molecules are lost or retained in the cross-linking process, γ-radiation sterilization, and sterilization with 70% ethanol. Nevertheless, all groups showed proliferation of cells over time, and the addition of fibrin glue and P4HB did not seem to affect the proliferation. Another limitation was that we chose to use primary vaginal fibroblasts from patients with pelvic organ prolapse (POP). Patients with POP have lower overall collagen content, decreased contractibility of (myo)fibroblasts, and an increase in collagen III, which is associated with increased flexibility and decreased tensile strength.^21,55^ We choose to use POP fibroblasts to be more representable of the disease model of vesicovaginal fistula, a notorious scarred and deprived environment, when compared to using fibroblasts derived from healthy volunteers. In addition, obtaining primary vaginal fibroblasts is challenging due to ethical issues for taking biopsies from healthy individuals.^54^ Harvesting fibroblasts from patients with fistula involves the same ethical difficulty, and vesicovaginal fistulas do not occur naturally in animals, excluding animal models as an alternative source for cells. Therefore, in the case of healthy cells, we would expect to achieve higher proliferation and collagen deposition, and outcomes in this study may underestimate the regenerative capacity of the VVF implant.^22^

## Conclusions

This study shows that a construct consisting of AM, fibrin glue, and electrospun P4HB is noninferior to AM alone for culturing of vaginal fibroblasts with good proliferation and migration of these cells on the constructs. P4HB contributes to the required mechanical strength of the construct and can be further modified for the intended application. This AM-based construct can be applied for vesicovaginal fistula repair in a large animal model or a pilot clinical trial to assess tissue integration and regeneration and functional outcomes of vesicovaginal fistula repair.^56^ An AM-based implant for surgical repair of internal organs requiring load-bearing functionality can be directly translated to other indications, e.g., gastric perforations, anastomotic leakage, urethral and penile reconstruction, bladder augmentation, cleft palate closure, tympanoplasty, pericardium closure, and closure of pleural membrane and nasal septum defects.

## Supporting Information Available

Fluorescent imaging day 14 and 28 (Figure S1); zymography (Figure S2); and mechanical characteristics (Table S1) (PDF)

## Abbreviations

AM: amniotic membrane
DMEM: Dulbecco’s modified Eagle medium
ECM: extracellular matrix
FBS: fetal bovine serum
FG: fibrin glue
GA: glutaraldehyde
MMP: matrix metalloproteinase
P4HB: poly-4-hydroxybutyrate
PBS: phosphate-buffered saline
SEM: scanning electron microscopy
VVF: vesicovaginal fistula
