# Supplementary material for: The Regenerative Capacity of Tissue-Engineered Amniotic Membranes
Source: ACS Appl Bio Mater. 2024 Feb 23;7(3):1441–8. doi: 10.1021/acsabm.3c00765 (PMC10951947; doi:10.1021/acsabm.3c00765)
Supplement: Supplementary file 1 — mt3c00765_si_001.pdf [file mt3c00765_si_001.pdf]

**SUPPLEMENTARY MATERIAL**

**Title: The regenerative capacity of tissue-engineered amniotic membranes**

Authors:
Lennart Maljaars [1,2],, Aksel Gudde [1,2], Anel Oosthuysen [3], Jan-Paul Roovers [1,2], Zeliha Guler [1,2*]

[1] Department of Obstetrics and Gynecology, Amsterdam UMC location University of Amsterdam, Meibergdreef 9, 1105 AZ Amsterdam, The Netherlands
[2] Amsterdam Reproduction and Development research institute, Meibergdreef 9, 1105 AZ Amsterdam, The Netherlands
[3] Cardiovascular Research Unit, University of Cape Town, Anzio Road, Observatory 7925, Cape Town, South Africa

*Corresponding author:
Z. Guler, Ph.D
Department of Obstetrics and Gynecology
Amsterdam Reproduction and Development
Amsterdam UMC, location AMC, University of Amsterdam
Meibergdreef 9, 1105 AZ Amsterdam, The Netherlands
Tel: +31 20 5666199
Zeliha.guler@amsterdamumc.nl

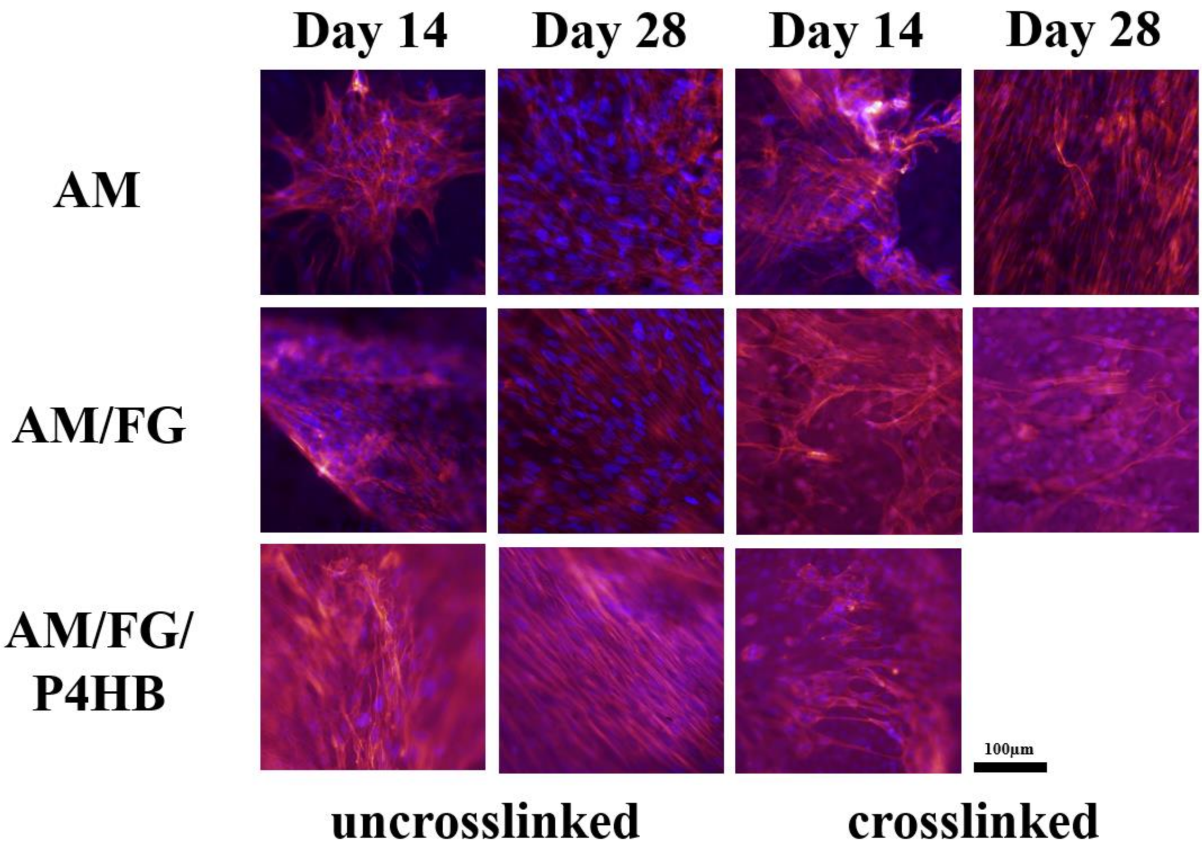


Supplementary figure 1 – Fluorescent imaging.
Immunofluorescence of vaginal fibroblast at culture day 14 and 28. crAM/FG/P4HB at day 28 was not available.

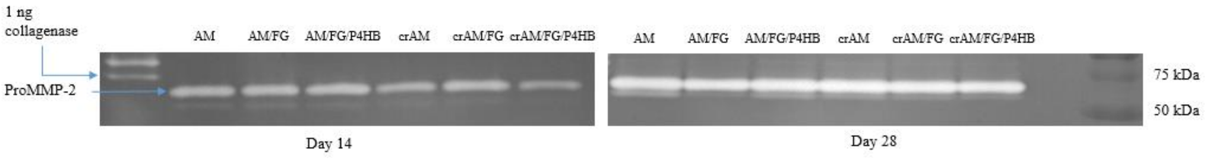


Supplementary figure 2: Zymography.

Example of zymography gel electrophoresis of proMMP-2 activity on day 14 and 28; on the left the reference categories with protein standard and 1ng collagenase. AM: uncrosslinked amniotic membranes; AM/FG: uncrosslinked amniotic membranes with fibrin glue; AM/FG/P4HB: uncrosslinked amniotic membranes with fibrin glue and electrospun poly-4-hydroxbutyrate (P4HB); crAM: crosslinked amniotic membranes; crAM/FG: crosslinked amniotic membranes with fibrin glue; crAM/FG/P4HB: crosslinked amniotic membranes with fibrin glue and electrospun poly-4-hydroxybutyrate (P4HB).

Supplementary table 1: Mechanical characteristics

| **Material** | | **Tensile stress at break (MPa)** | **Maximum elongation (%)** | **Tangent modulus (MPa)** | **Maximum load (N)** |
|---|---|---|---|---|---|
| Amniotic membranes | dry | 10.5 (±3.9) | 2.5 (±1.2) | 5.63 (±2.50) | 1.1 (±0.4) |
| | wet | 3.8 [2.7-10.4] | 12.5 (±3.4) | 0.81 (±0.57) | 0.4 [0.3-1.0] |
| P4HB (control) | P4-T0 | 4.5 (±0.5) | 112.3 (±12.2) | 0.31 (±0.03) | 19.1 (±1.8) |
| | P4-T4 | 3.5 (±0.8) | 74.7 (±26.1) | 0.48 (±0.05) | 14.7 (±3.5) |
| | P4-T8 | 3.6 (±0.4) | 64.0 (±15.6) | 0.62 (±0.11) | 16.5 (±2.5) |
| P4HB + fibrin glue | P4FT0 | 2.7 (±0.3) | 66.6 (±7.2) | 0.23 (±0.02) | 13.7 (±1.4) |
| | P4FT4 | 1.6 (±0.5) | 24.1 (±12.8) | 0.37 [0.36-0.45] | 6.6 (±1.6) |
| | P4FT8 | 0.8 (±0.3) | 9.4 (±2.6) | 0.29 (±0.06) | 4.0 (±1.6) |
| P4HB + fibrin glue + AM | P4AT0 | 2.8 (±0.2) | 70.1 (±5.6) | 0.21 (±0.02) | 14.6 (±1.2) |
| | P4AT4 | 2.6 (±0.2) | 59.4 (±2.0) | 0.37 (±0.05) | 12.2 (±0.7) |
| | P4AT8 | 1.0 (±0.2) | 12.5 (±2.4) | 0.30 (±0.08) | 5.0 (±0.8) |

Data is reported as mean (±standard deviation) or median [$25^{th}$ – $75^{th}$ percentile]; All samples had width of 10mm;
*AM*: amniotic membrane; *P4HB*: poly-4-hydroxybutyrate; cxl: crosslinking experiment; P4-: P4HB control sample; P4F: P4HB with fibrin glue; P4A: P4HB with AM glued together with fibrin glue; T0: baseline measurement; T4: degradation 4 weeks; T8: degradation 8 weeks;

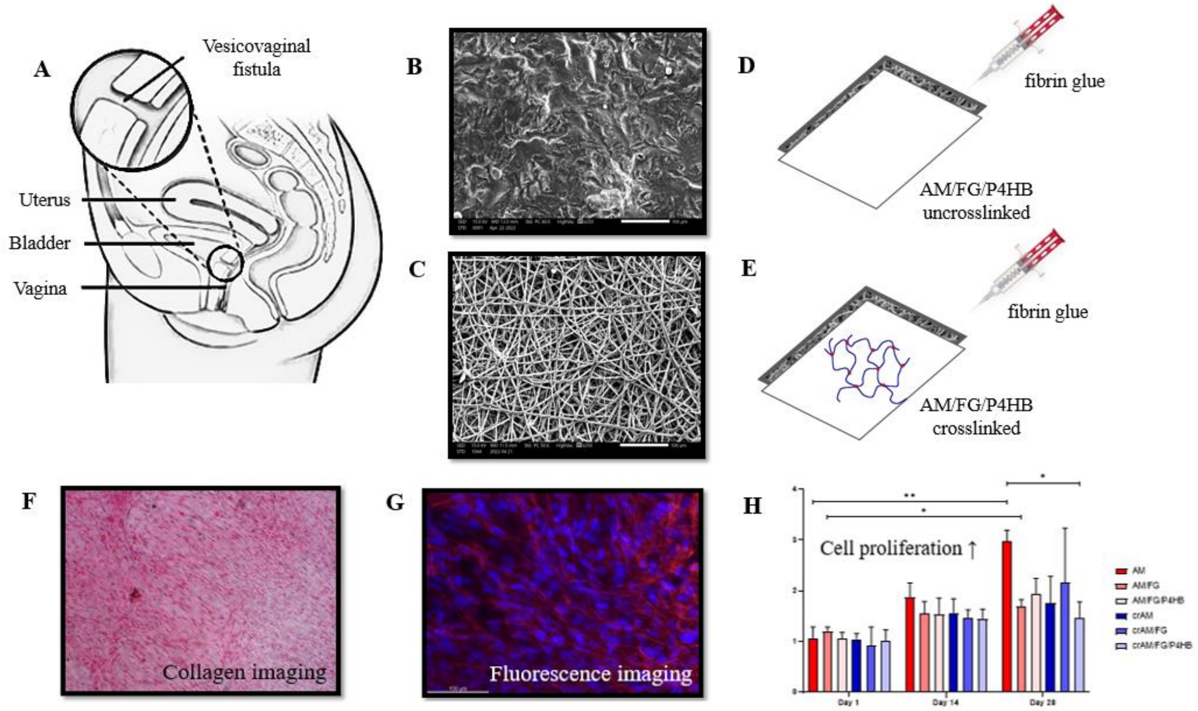


A: Schematic representation of a vesicovaginal fistula; B: Scanning electron microscopy (SEM) image of amniotic membranes at ×250-magnification; C: SEM image of poly-4-hydroxybutyrate (P4HB) at ×250-magnification; D: Preparation of P4HB samples: P4HB scaffold and uncrosslinked AM (white sheet) glued together with fibrin glue; E: Preparation of P4HB samples: P4HB scaffold and crosslinked AM (white sheet with crosslinking network) glued together with fibrin glue; F: Example of collagen imaging; G: Example of fluorescence imaging; H: Graph with cell proliferation data.
